# Fungal transformation of androsta-1,4-diene-3,17-dione by *Aspergillus brasiliensis*

**DOI:** 10.1186/s40199-014-0071-8

**Published:** 2014-11-15

**Authors:** Tahereh Hosseinabadi, Hossein Vahidi, Bahman Nickavar, Farzad Kobarfard

**Affiliations:** Department of Pharmacognosy and Biotechnology, School of Pharmacy, Shahid Beheshti University of Medical Sciences, Vali-e Asr Ave., Niayesh Junction, Tehran, 1996835113 Iran; Department of Medicinal Chemistry, School of Pharmacy, Shahid Beheshti University of Medical Sciences, Vali-e Asr Ave., Niayesh Junction, Tehran, 1996835113 Iran

**Keywords:** *Aspergillus brasiliensis*, Fungi, Androsta-1, 4-diene-3, 17-dione, Steroid, Biotransformation

## Abstract

**Background:**

The biotransformation of steroids by fungal biocatalysts has been recognized for many years. There are numerous fungi of the genus *Aspergillu*s which have been shown to transform different steroid substances. The possibility of using filamentous fungi *Aspergillus brasiliensis* cells in the biotransformation of androsta-1,4-diene-3,17-dione, was evaluated.

**Methods:**

The fungal strain was inoculated into the transformation medium which supplemented with androstadienedione as a substrate and fermentation continued for 5 days. The metabolites were extracted and isolated by thin layer chromatography. The structures of these metabolites were elucidated using ^1^H-NMR, broadband decoupled ^13^C-NMR, EI Mass and IR spectroscopies.

**Results:**

The fermentation yielded one reduced product: 17β-hydroxyandrost-1,4-dien-3-one and two hydroxylated metabolites: 11α-hydroxyandrost-1,4-diene-3,17-dione and 12β-hydroxyandrost-1,4-diene-3,17-dione.

**Conclusions:**

The results obtained in this study show that *A. brasiliendsis* could be considered as a biocatalyst for producing important derivatives from androstadienedione.

## Background

Microbial biotransformation by the whole cell microorganisms is economically and ecologically a competitive tool for the biotechnological professionals in search of new techniques to manufacture valuable chemicals, pharmaceutical and agrochemical compounds [1,2]. The production of steroid drugs and hormones is one of the best examples of the successful application of microbial technology in large scale industrial processes [3]. A large number of bacterial and fungal species are able to bio-transform steroid compounds [4]. Among them, there are numerous fungi of the genus *Aspergillu*s including *A. wentti, A. niger, A. nidulans, A. ochraceus*, *A. parasiticus, A. oryzae*, *A. flavu*s, *A. tamari, A. parasiticus* and *A. fumigatus* which have been used for the biotransformation of many steroids and shown to mediate hydroxylation, oxidation, reduction, double bond formation and epoxidation of various steroid substances [5-7]. Insertion of a hydroxyl group to a steroid molecule is one of the most important steps in the production of steroidal derivatives which is carried out by many of filamentous fungi. Several positions in the steroid molecules can be hydroxylated by various microbial strains via their hydroxylase enzyme [8].

The filamentous fungi, *Aspergillus brasiliensis* is a biseriate black species, described and named within *Aspergillus* section *Nigri*, by Varga et al. in 2007. It is differentiable from the other black aspergilla because of its unique morphology, extrolite profiles and genotypic features [9]. Literature review shows that there is no report indicating the ability of this filamentous fungus to modify the structure of steroids. In the present work, the capability of *A. brasiliensis* was evaluated for the biotransformation of androsta-1,4-diene-3,17-dione (ADD) as an exogenous substrate. ADD is one of the most important steroids, which is used as a precursor for preparing some pharmaceutically-interesting steroids. It is commercially produced by the microbiological transformation of β-sitosterol and cholesterol [8]. It is presently used in the industrial synthesis of estradiol or estrone [4,10].

Biotransformation of this substrate, has already been reported by some other fungi, such as *Mucor racemosus, Acremonium strictum*, *Cephalosporium aphidicola* and *Neurospora crassa* leading to the production of different compounds [11-14].

## Experimental

### Materials

ADD was purchased from Sigma- Aldrich. All other chemicals and reagents used, were of analytical grade and commercially available.

### Microorganism

The fungal strain *A. brasilliensis* PTCC 5298 was purchased From Iranian Research Organization for Science and Technology (IROST).

Cultures of fungi were grown at 26°C for 5 days until good sporulation was obtained on Czapec medium, consisting of 30 g sucrose, 2.0 g NaNO_3_, 1.0 g K_2_HPO_4_, 0.50 g MgSO_4_, 0.50 g KCl, 0.01 g FeSO_4_, 15 g Agar and 1000 ml DW, based on IROST catalogue for this fungus [15]. Stock cultures were maintained at 4°C on Czapec medium slopes and freshly subcultured before use in transformation experiments. The organism was transferred to fresh medium and refreshed every two weeks.

### Inoculum preparation and biotransformation process

Spores freshly obtained from Czapec slopes were washed with distilled water (DW) containing Tween-80 and transferred aseptically into 500 ml flasks containing 100 ml sterile medium, in a biological safety cabinet (pH of the medium was adjusted to 7.4 before sterilization).

Volume of inoculums, containing 1 × 10^6^ spores, was used in all experiments unless otherwise stated. After cultivation at 26°C for 2 days on a rotary shaker (125 rpm) and pellet formation, ADD (100 mg) was dissolved in 1 ml acetone and aseptically added to each flask. A parallel control without substrate and also a culture medium, containing substrate but no microorganism were run concurrently (as control cultures). Biotransformation was carried out under above condition for further 5 days.

Sampling was carried out every 24 h. The samples were extracted with three volumes of chloroform and the transformation was then checked using thin layer chromatography (TLC).

After detecting the transformation on TLC plate, the fermentation was conducted on the larger scale.

Ten 1000 ml-Erlenmeyer flasks were filled with 200 ml cultivation medium. The culture media were incubated under the same conditions and then 1000 mg of substrate, (dissolved in 10 ml acetone) was distributed evenly among the flasks and process continued for 5 days.

All the experiments were performed in duplicate.

### Product isolation and analyses

At the end of incubation, the fungus mycelium was separated from the broth by filtration and the mycelium was rinsed with DW. Mycelia and the filtrate were separately extracted with chloroform (3 volumes), dried over anhydrous sodium sulfate and concentrated under vacuum. The residue was analyzed by TLC, then loaded on chromatography plates and fractionated with chloroform/acetone (6.5:3.5 v/v) as the eluent solvent. The metabolites were then separated from silica gel using a mixture of methanol/chloroform/acetone (three times). The transformation products were analyzed and identified using different spectroscopic data (^13^C NMR, ^1^H NMR, FTIR and MS).

### Instruments

Melting points (mp) were determined on thermoscientific 9200 apparatus and were uncorrected.

^1^H and ^13^C nuclear magnetic resonances (NMR) spectra were recorded using a Bruker DRX (Avance 500) spectrometer (Rheinstetten, Germany) at 500 and 125 MHz, respectively, in CDCl_3_ with tetramethylsilane (TMS) as the internal standard. Chemical shifts (δ) are given in parts per million (ppm) relative to TMS. The coupling constants (J) are given in hertz (Hz). Infrared (IR) spectra were recorded on a Perkin-Elmer 843 spectrometer with KBr as a diluent. Mass spectra (MS) were obtained using Agilent 6410 Triple Quadrupole mass spectrometer. TLC was conducted on 0.25 mm thick layers of silica gel G (Kieselgel 60 HF_254+366_, Merck). Chromatography plates were developed with chloroform/acetone (3.5:6.5, v/v) and visualized by spraying the plates with a mixture of methanol/ sulfuric acid (6:1, v/v) and heating them in an oven at 100°C for 3 min until the colors developed. The compounds were also visualized under a UV lamp (Strstedt– Gruppe HP-UVIS) at 254 nm.

## Results

**Figure 1:**
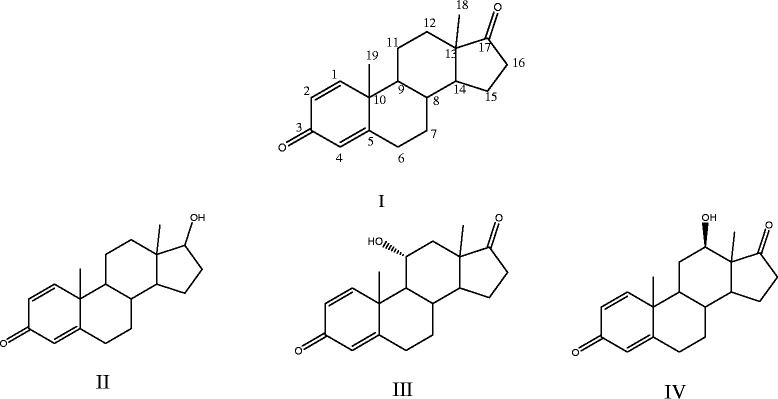
**The structure of androstadienedione (I) and its metabolites; 17β-Hydroxyandrost-1,4-diene-3-one (II), 11α-Hydroxyandrost-1, 4-diene-3,17-dione (III) and 12β-Hydroxyandrost-1,4-diene -3,17-dione (IV).**

Microbial transformation of ADD by *A. brasiliensis* in 5 days resulted in the formation of three hydroxysteroid-1,4-dien-3-one derivatives (II to IV), presented in Figure 1. No transformation occurred in the control media. Steroid products were characterized using different spectroscopic data (^13^C NMR, ^1^H NMR, FTIR and MS) and melting points.

The analytical data for compounds II–IV are mentioned in a respective order. ^13^C NMR assignments for the substrates and metabolites are listed in Table 1.

**Table 1:** ^**13**^ **C NMR data determined in CDCl**_**3**_ **at 500 MHz for compounds I-IV**

**Carbon atom**	**I**	**II**	**III**	**IV**
1	155.20	156.00	158.81	155.35
2	127.60	127.46	128.80	127.66
3	186.00	186.45	186.77	186.28
4	124.08	123.83	124.71	124.10
5	168.19	169.30	167.66	168.33
6	32.29	32.78	32.85	32.48
7	31.21	33.14	29.70	33.32
8	35.08	35.56	33.96	34.35
9	52.31	52.44	60.48	52.13
10	43.39	43.6	43.0	43.48
11	22.07	22.50	67.69	36.03
12	32.51	36.30	42.29	85.97
13	47.62	43.1	47.85	42.64
14	50.41	50.09	49.59	44.13
15	21.88	23.53	21.85	22.16
16	35.58	30.32	35.79	35.53
17	219.66	81.41	218.25	216.31
18	13.80	11.17	14.59	11.50
19	18.70	18.72	18.65	18.72

### 17β-hydroxyandrost-1, 4-diene-3-one (Boldenone) (II)

Colorless crystalline compound; yield 24.5%; mp 169–172°C; R_f_ (acetone/chloroform 3.5:6.5, v/v): 0.71; IR *ν*_max_ 3489, 1666, 1619 cm^−1^; MS (EI) m/z: 286 (M^+^, C_19_H_26_O_2_), 227, 159, 147, 121, 91, 77; ^1^H NMR (CDCl_3_, 500 MHz) *δ* 7.07 (1H, d, J = 10 Hz, H-1), 6.22 (1H, d, J = 10 Hz, H-2), 6.07 (1H, s, H-4), 3.64 (1H, t, J_17α,16αβ_ = 8.5 Hz, H-17α), 2.06 (1H, m, H-16α), 1.55 (1H, m, H-16β), 1.24 (3H, s, H-19), 0.82 (3H, s, H-18); ^13^C NMR (CDCl_3_) *δ* 186.4 (C-3), 169.3 (C-5), 156.0 (C-1), 127.5 (C-2), 123.8 (C-4), 81.4 (C-17), 18.7 (C-19), 11.1(C-18).

### 11α-hydroxyandrost-1,4-diene-3,17-dione (III)

Colorless crystalline compound; yield 9.6%; mp 210–214°C; R_f_ (acetone/chloroform 3.5:6.5, v/v): 0.29; IR *ν*_max_ 3456, 1737, 1663, 1622 cm^−1^; MS (EI) m/z: 300 (M^+^, C_19_H_24_O_3_), 282 , 231, 161, 124, 109, 84, 55; ^1^HNMR (CDCl_3_, 500 MHz): *δ* 7.27 (1H, d, J = 10 Hz, H-1), 6.15 (1H, d, J = 10 Hz, H-2), 6.08 (1H, s, H-4), 4.12 (1H, m, H-11_β_), 2.31(1H, m, H-12_β_), 1.54 (1H, m, H-12_α_), 1.24 (3H, s, H-19), 0.96 (3H, s, H-18); ^13^CNMR (CDCl_3_) *δ* 218.2 (C-17), 186.7 (C-3), 167.6 (C-5), 158.8 (C-1), 128.8 (C-2), 124.7 (C-4), 67.69 (C-11), 18.6 (C-19), 14.5 (C-18).

### 12β-hydroxyandrost-1,4-dien-3,17-dione (IV)

Colorless crystalline compound; yield 10.3%; mp 164–167°C; R_*f*_ (acetone/chloroform, 3.5:6.5 v/v): 0.60; IR *ν*_max_ 3408, 1741, 1661, 1618 cm^−1^; MS (EI) m/z 300 (M^+^, C_19_H_24_O_3_), 161, 147, 134, 122, 91, 55; ^1^H NMR (CDCl_3_, 500 MHz) δ 7.06 (1H, d, J = 10 Hz, H-1), 6.24 (1H, d, J = 10 Hz, H-2), 6.07 (1H, s, H-4), 3.74 (1H, s, H-12), 2.03, 1.45 (1H, m, H-11), 1.25 (3H, s, H-19), 0.79 (3H, s, H-18); ^13^C NMR (CDCl_3_) *δ* 216.3 (C-17), 186.3 (C-3), 168.3 (C-5), 155.3 (C-1), 127.6 (C-2), 124.1 (C-4), 85.97 (C-12), 18.7 (C-19), 11.50 (C-18).

### Spectra interpretation

The EI-MS spectrum of compound II showed the M^+^ at m/z 286 which corresponds to the molecular formula C_19_H_26_O_2_, 2 a.m.u. higher than the molecular weight of parent compound and thus indicated a possible hydrogenation of compound I. The IR spectrum showed an absorbance at 3489 cm^- 1^, characteristic of a hydroxyl group. The lack of absorption band at 1736 cm^−1^ (17-ketone) and the existence of a peak at 3489 cm^−1^, verify the reduction of the carbonyl group to a hydroxyl group at C-17 position. The ^1^H-NMR spectrum of II showed an additional methine proton signal at δ 3.64 that is assigned to 17-H. This modification is confirmed by the appearance of a new methine carbon signal at δ 81.41 in ^13^C-NMR spectra. The stereochemistry of the newly formed hydroxyl group was deduced to be β, based on the chemical shift of 17-H (δ 3.64), its coupling constant (J = 8.5 Hz) and splitting pattern (triplet) which is in agreement with the published data for a C-17α proton in 17β-hydroxysteroids [13,16]. This pattern is often seen when the 17β position is substituted with a hydroxyl group (e.g. pregnan-20-ones7 or androstan-17β-ols) [17]. In the case of 17α-hydroxy steroids, the splitting of H-17 β is generally a doublet [18].

The EI-MS spectrum of the transformed product III, showed the M^+^ at m/z 300 (calcd for C_19_H_24_O_3_ 300.1749), which was 16 a.m.u. higher than the molecular weight of compound I, thus suggesting the possible hydroxylation of it. The IR spectrum displayed hydroxyl signal at 3456 cm^−1^. ^1^H- and ^13^C-NMR spectra were very similar to those of the substrate, except for a new downfield methine proton signal at δ 4.12 (ddd, J_11β,12β_ = 12.2 Hz, J_11β,9α_ = 10.3 Hz, J_11β,12α_ = 5.4 Hz) which was assigned to the methine H-11 geminal to OH and with a downfield methine carbon signal at δ 67.69. This finding is also confirmed by the data reported in literature for this compound [13].

The mass spectrum for compound IV, showed the molecular ion peak at *m*/*z* 300 (C_19_H_24_O_3_), which suggested the possible insertion of one oxygen atom in the structure of the substrate (I). The IR spectrum, showed two carbonyl absorption bands at 1741 and 1661 cm^−1^ for C-17 and C-3 respectively. The absorbance at 3408 cm^−1^, confirmed the existance of a hydroxyl group. Melting point, ^13^C-NMR and ^1^H-NMR spectral data, assignments and chemical shifts for this compound were in agreement with those which have been reported by Zafar et al. in 2013 for 12β-hydroxyandrost-1,4-dien-3,17-dione [19].

## Discussion

In light of the results obtained in this study, it appears that the *A. brasiliensis* transformation of ADD led to the formation of three major bioproducts. The bioconversion characteristics observed were 17-ketone reduction, 11α and 12β-hydroxylation.

ADD is one of the important intermediates for producing some valuable pharmaceutical steroid compounds and has been used in many studies as a substrate of the biotransformation experiments [20]. Compound II, Boldenone, also called 1-dehydrotestosterone or androsta-1, 4-dien-17β-ol-3-one, is a steroid which only differs from testosterone by one double bond at position 1. Reduction of 17-keto group of ADD results in formation of boldenone. Boldenone with its low androgenic characteristics but strong anabolic characteristics allows improving anabolic processes like growth and development of muscle mass without any undesired side-effects [21].

Reduction of 17-carbonyl group of ADD has been reported previously by *Mucor racemosus* and *Acremonium strictum* and *Cephalosporium aphidicola* fermention [11,12]. 11α-hydroxyandrost-1,4-diene-3,17-dione (III) which is used in the preparation of anti-osteoporosis active compounds, has been previously obtained by ADD biotransformation in *Cephalosporium aphidicola, Rhizopus arrhizus* and *Aspergillus ochraceus* culture media [13,20,22]. It has also been reported by fermentation of dihydrotestosterone (DHT) with *Gibberella fujikuroi*. This compound which is significant and specific inhibitor of butyrylcholinesterase (BChE), in comparison to standard drug, galanthamine. 12β-hydroxyandrost-1,4-diene-3,17-dione (IV) was also another hydroxylated steroid which has been obtained from DHT biotransformation by *G. fujikuroi* [19]. Although hydroxylation of steroidal substrates is common by filamentous fungi, hydroxylation at C-12 position is relatively rare [6]. The 12β-hydroxylation is proprietary reaction for filamentous fungi and is unknown in humans [23]. Introduction of a C-12 substituent and especially a β C-12 substituent into glucocorticoids improves their usefulness as topical anti-inflammatories by increasing their topical activity relative to their systemic activity [24]. Therefore from an industrial viewpoint, the ability of *A. brasiliensis* to carry out 12β-hydroxylation on ADD substrate may be interesting as a process for production of glucocorticoids.

## Conclusion

The present research shows that the transformation of androstendienedione using *A. brasiliensis* whole cells yielded interesting transformation products. The observed modifications included hydroxylation at C-11α, C-12β and 17-carbonyl reduction into the related C-17β hydroxyl forms. These products were separated and characterized on the basis of their spectral data. To the best of our knowledge, there are only few reports for ADD biotransformation by microorganisms and also no report for *A. brasiliensis.* Therefore *A. brasiliensis* could be considered as efficient biocatalyst for preparation of new steroids with commercial significance.

## Abbreviations

ADD: Androsta-1,4-diene-3,17-dione
DHT: Dihydrotestosterone
TLC: Thin layer chromatography
NMR: Nuclear magnetic resonances
